# The Role of Substrate Surface Geometry in the Photo-Electrochemical Behaviour of Supported TiO_2_ Nanotube Arrays: A Study Using Electrochemical Impedance Spectroscopy (EIS)

**DOI:** 10.3390/molecules28083378

**Published:** 2023-04-11

**Authors:** Luana De Pasquale, Francesco Tavella, Victor Longo, Marco Favaro, Siglinda Perathoner, Gabriele Centi, Claudio Ampelli, Chiara Genovese

**Affiliations:** 1 Department of Chemical, Biological, Pharmaceutical and Environmental Sciences, University of Messina, ERIC aisbl and CASPE/INSTM, V.le F. Stagno d’Alcontres 31, 98166 Messina, Italy; 2 Institute for Solar Fuels, Helmholtz-Zentrum Berlin für Materialien und Energie GmbH, Hahn-Meitner-Platz 1, 14109 Berlin, Germany

**Keywords:** electrochemical impedance spectroscopy, Ti mesh, 3D nanostructures, H_2_ production

## Abstract

Highly ordered TiO_2_ nanotube (NT) arrays grown on Ti mesh and Ti foil were successfully prepared by a controlled anodic oxidation process and tested for water photo-electrolysis. Electrochemical impedance spectroscopy (EIS), combined with other electrochemical techniques (cyclic voltammetry and chronoamperometry) in tests performed in the dark and under illumination conditions, was used to correlate the photoactivity to the specific charge transfer resistances associated with a 3D (mesh) or 2D (foil) geometry of the support. The peculiar structure of the nanotubes in the mesh (with better light absorption and faster electron transport along the nanotubes) strongly impacts the catalytic performances under illumination. H_2_ production and current density in water photo-electrolysis were over three times higher with the TiO_2_NTs/Ti mesh, compared to the foil in the same conditions. The results obtained by the EIS technique, used here for the first time to directly compare TiO_2_ nanotubes on two different supports (Ti foil and Ti mesh), led to a better understanding of the electronic properties of TiO_2_ nanotubes and the effect of a specific support on its photocatalytic properties.

## 1. Introduction

Photo-electrocatalytic (PEC) technology for hydrogen production is an intriguing, environmentally friendly approach for directly converting solar energy into fuels [1]. TiO_2_-based catalysts are still largely used as photocatalysts and for H_2_ production by water photo-electrolysis. Anodic oxidation of titanium foil has become one of the popular approaches for obtaining a nanostructured array of TiO_2_ film. As photoanode for H_2_ production by visible light, many efforts have been made to improve the photoactive performances of the materials, typically by creating heterojunctions or adding cocatalysts for tailoring the catalytic performances of the semiconductors. In the design of efficient photocatalysts, a three-dimensional ordered TiO_2_ nanotube array grown on a mesh has a leading role, due to its larger internal and external surface areas, which can harvest the light from any direction and improve the activity in water photo-electrolysis. It was demonstrated that the nanotubes formed on a Ti wire could absorb incident, reflected and refracted light in all directions, minimizing the liquid’s scattering effects and increasing the photoactivity [2,3,4,5,6,7].

As we earlier demonstrated, developing photoactive materials with novel 3D-type structural characteristics guaranteed good properties of both light harvesting and charge transport, due to the combination of a mesoporous structure (due to the ordered array of TiO_2_ nanotubes) with the macro pores of the gauze [8]. In a photo-(electro)catalytic process, it becomes essential to understand the local phenomena that affect the process itself, related to the transport of charges on the semiconductor, to the redox reactions at its surface and to mass transfer phenomena (ionic migration) in the liquid electrolyte (if present,) particularly occurring at the interface between electrode and liquid.

Among the electrochemical characterization techniques available, electrochemical impedance spectroscopy (EIS) is one of the most useful for investigating the electrical behaviour of photo and electrocatalytic systems, thanks to the apparent easiness of execution, [9] even if the interpretation of the experimental plots is sometimes complex, requiring an accurate fitting to an equivalent electrical circuit model (ECM) consisting of common electrical components (capacitors, inductors resistors, etc.) [10,11].

In general terms, this method consists of measuring the overall response of an electrode (depending on several processes that simultaneously occur on the surface of the conductive material and at the electrolyte) to a small sinusoidal alternate current (AC) at different frequencies at a fixed applied potential [12].

The use of EIS applied to semiconductor electrodes such as TiO_2_ photocatalysts (in the form of nanotube arrays or porous thin film) is a common procedure reported in several works [13,14,15,16,17,18], but this is the first time, as far as we know, that the EIS has been used as a tool to investigate how a different geometry can influence the electrochemical properties of materials providing insight into the activity in hydrogen evolution reaction (HER). Only a few studies reported EIS applied to TiO_2_ nanotube arrays on a mesh [19,20,21]. The present study aims to investigate the effect of different geometrical support (foil vs. mesh) on the electrical transport properties of TiO_2_ nanotube arrays deposited by anodization synthesis to obtain insights into how charge transfer phenomena can influence photocatalytic activity. No other papers in the literature have investigated this specific aspect.

For this investigation, highly ordered TiO_2_ nanotubes (NTs) grown on Ti mesh and Ti foil were prepared using controlled anodization. H_2_ production in water photo-electrolysis was significantly enhanced by using a 3D nanostructure. The correlation between the different individual charge transfer resistances associated with the substrate surface geometry (3D as mesh or 2D as foil) was analyzed under dark and illuminated conditions. EIS results were complemented by cyclic voltammetry (CV) and chronoamperometry (CA) studies. The results provide new insight into the design and fabrication of highly efficient photocatalysts, helping to understand the role of substrate surface geometry in water photo-electrolysis [22], an aspect not clarified before.

## 2. Results and Discussion

### 2.1. XRD and SEM Results

The phase composition of the TiO_2_NTs-based photocatalysts was investigated using XRD. The diffraction patterns are reported in Figure S1 in the Supplementary Info (SI). All the diffraction peaks belong to the crystal structure of anatase TiO_2_ (JCPDS 00-021-1272), showing a high crystallinity grade for TiO_2_NTs on both Ti foil and mesh. No rutile or other titania phases were detected in the two samples. The XRD patterns indicate the presence of metallic Ti, related to the substrate on which the TiO_2_NTs are grown during the anodization process. It gives robustness to the electrode and acts as a conductive electron collector.

**Figure 1:**
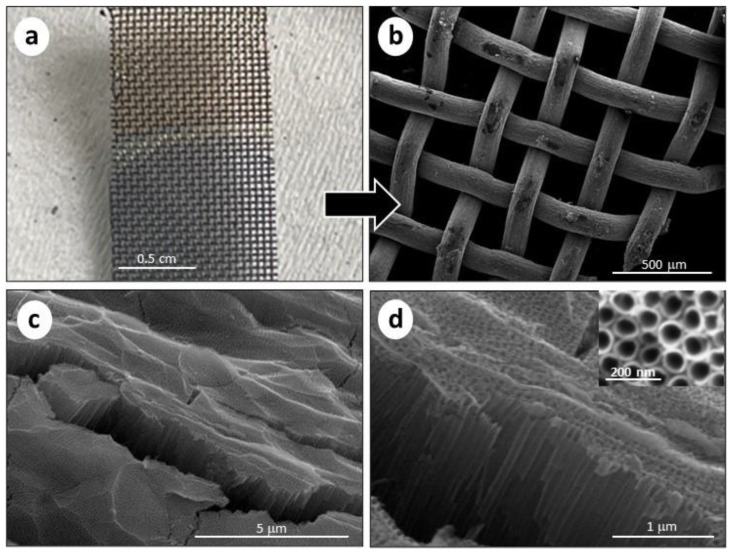
(**a**) Picture of the Ti mesh before (upper half) and after (bottom half) the anodization, with a colour difference; (**b**) SEM image of the TiO_2_NTs/Ti mesh after the anodization (50 V, 1 h); (**c**) cross-section SEM image of TiO_2_NTs/Ti mesh at low magnification and (**d**) high magnification. The insert in (**d**) refers to the top view of the nanotube arrays.

The morphological characteristics of the TiO_2_NTs were investigated using SEM analysis. The synthesis parameters (i.e., applied voltage and anodization time) were optimized during experimentation on the flat Ti foils [8,23] and applied to the Ti mesh substrate. Figure 1 shows the SEM images for TiO_2_NTs on Ti mesh, prepared at 50 V in 1 h of anodization time. Elemental analysis obtained by EDX is reported in Figure S2 in SI.

A photograph of the Ti mesh is also reported (see Figure 1a), showing the oxidation of the substrate, which changes its colour. The macro-structure was maintained, compared to the non-oxidized Ti mesh (see Figure S3 in SI). Nevertheless, the surface is roughened, due to the formation of the titania nanotubes grown on the round surface of the woven Ti wires. Figure 1c,d show the cross-section SEM images of the TiO_2_NTs at different magnifications, evidencing the presence of vertically aligned TiO_2_NTs with an average length of 1.5 µm. The inner diameter of the TiO_2_NTs was 70–80 nm, as directly measured from the top-view image (see the insert in Figure 1d). Due to the macro holes of the meshed structure, the resulting TiO_2_NTs on the Ti mesh have an open area of about 33%. Figure S4 in SI shows SEM images of the TiO_2_NTs on Ti foil, evidencing a nanotube length of 1.4 μm and 60–70 nm diameter. Thus, both TiO_2_NTs on Ti mesh and Ti foil show similar morphological characteristics, except for the specific 3D macrostructure of the mesh compared to the foil.

### 2.2. Optical Absorption Properties and Photocurrent Response

The TiO_2_NTs/Ti, mesh and foil electrodes were characterized using UV-Vis diffuse reflectance spectroscopy to evaluate their optical absorption properties. The spectra are shown in Figure S5 in SI. The spectrum of TiO_2_ P25 (a widely used reference material) is also reported for comparison. Both the profiles show an absorption peak below 400 nm, due to the typical UV light response of Ti dioxide correlated to the lowest energy charge transfer O^2−^/Ti^4+^. Moreover, intense, broad light absorption in the visible region (between 500 and 1200 nm) is present, due to light diffraction and scattering caused by nanotube arrays and defects. This absorption band is broader for the TiO_2_NTs deposited on Ti mesh for a more complex nanoarchitecture in a 3D structure with respect to the planar-type TiO_2_NTs/Ti foil [8].

**Figure 2:**
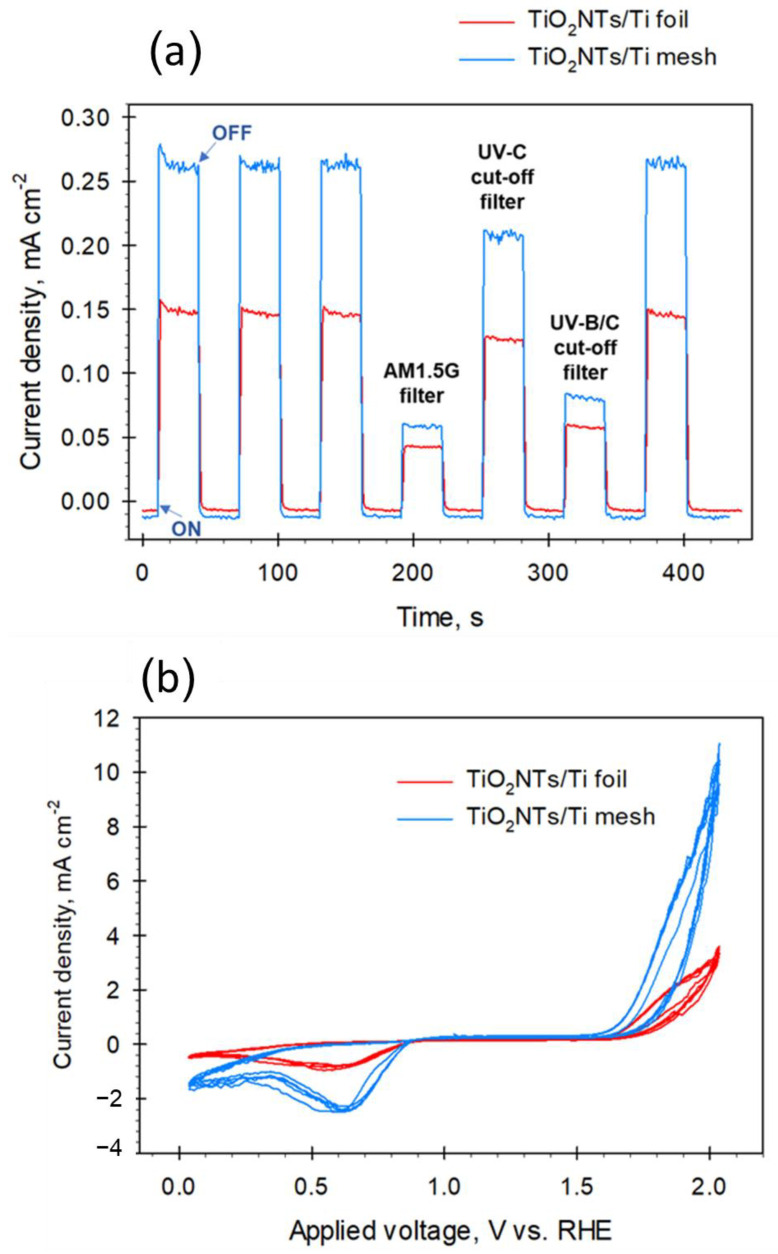
(**a**) Chronoamperometric measurements for TiO_2_NTs/Ti mesh and TiO_2_NTs/Ti foil electrodes (1.136 V vs. RHE, 1 M KOH) using open UV-visible lamp spectrum (no light filter) and with light filter (AM1.5G, UVC, and UVB/C blocking filter); (**b**) Cyclic voltammetry for the same electrodes in 1 M KOH. Data were normalized with respect to the surface area of the catalysts.

To evaluate the photo-current performances of TiO_2_NTs/Ti mesh compared to TiO_2_NTs/Ti foil, chronoamperometric (CA) experiments were performed at +1.136 V vs. RHE in 1 M KOH aqueous solution. The tests were carried out by applying ON/OFF illumination cycles in the presence of cut-off light filters (i.e., AM1.5 G, UVC blocking filter, UVB/C blocking filter). The data are shown in Figure 2a. Stability tests over TiO_2_NTs/Ti mesh showed a steady-state current density until 2 h under light irradiation (see Figure S6 in SI).

The first three cycles (and the last one) were performed at open spectrum (no light filters), showing good reproducibility behaviour, with a quick rise in photocurrent and fast recovery to the original value. The baseline (i.e., dark values) seems slightly lower for the mesh (less than 0.01 points), due to the higher resistance of the woven wires of the gauze with respect to a planar foil. However, the photocurrent density response of the TiO_2_NTs/Ti mesh was 1.7 times higher than that of TiO_2_NTs/Ti foil at open spectrum, indicating a better photocurrent, possibly associated with a lower charge recombination. Note that both the photo-electrodes were still active after filtering parts B and C of the UV spectrum (leaving part A of the UV and visible regions) and simulating the standard terrestrial solar irradiation with the AM1.5G filter (with the UV part only about 4% of the entire spectrum). On the other hand, unstructured TiO_2_ anatase films prepared from TiO_2_ powder show an almost negligible photocurrent when the AM 1.5 G filter is applied [24,25]. Thus, the TiO_2_NTs nanostructure favours a partial activity with visible light, because the decrease in the photocurrent is less marked than expected if only the 4% UV component allows the creation of the charge separation.

Figure 2b shows the cyclic voltammetry (CV) analysis performed on the same electrodes. The intensities of the peaks confirmed what was already observed by the CA investigation, i.e., a higher photo-current generated by the TiO_2_NTs/Ti mesh with respect to the foil, with an onset for water oxidation at about 1.5 V vs. RHE for both the electrodes. Note also that there is an intensification of the negative peak, with a maximum of around 0.6 V, not present in pure titania [26], and associated with the reaction of TiO_2_ with the electron/proton forming TiOOH on defective sites. It may also be noted that in TiO_2_NTs/Ti mesh there is a decrease in the voltage gap between the onsets of hydrogen and oxygen evolution reactions with respect to TiO_2_NTs/Ti foil. The gap passes from around 1.8 V to 1.2 V, thus indicating a significant reduction in the overpotential for water electrolysis. Chronoamperometric experiments and cyclic voltammetry performed on TiO_2_ P25 in the same conditions, are reported in Figure S7 in SI, as a comparison. As expected, the photoactivity is much lower compared to nanostructured electrodes.

### 2.3. EIS Studies

Electrochemical impedance spectroscopy (EIS) is a useful methodology to evaluate charge transfer phenomena occurring at the interface of electrodes/electrolyte [27]. EIS was recently performed over TiO_2_ nanotube arrays grown on Ti foils, to study the photo-induced phenomena and measure the charge current density under different operating conditions (e.g., electrolyte, pH, presence of dopants, etc.). In a few papers, EIS analysis has also been adopted to study the electrochemical behaviour of TiO_2_ nanotube arrays grown on Ti mesh [19,20,28,29].

EIS is based on applying a sinusoidal voltage with variable frequency to the system under investigation, measuring the current response to this perturbation. EIS data are typically fitted through an equivalent electrical circuit composed of elements (resistances, capacitances, etc.), mimicking the electrical behaviour of the system under study. The fitting gives quantitative information about the processes involved in the reaction.

**Figure 3:**
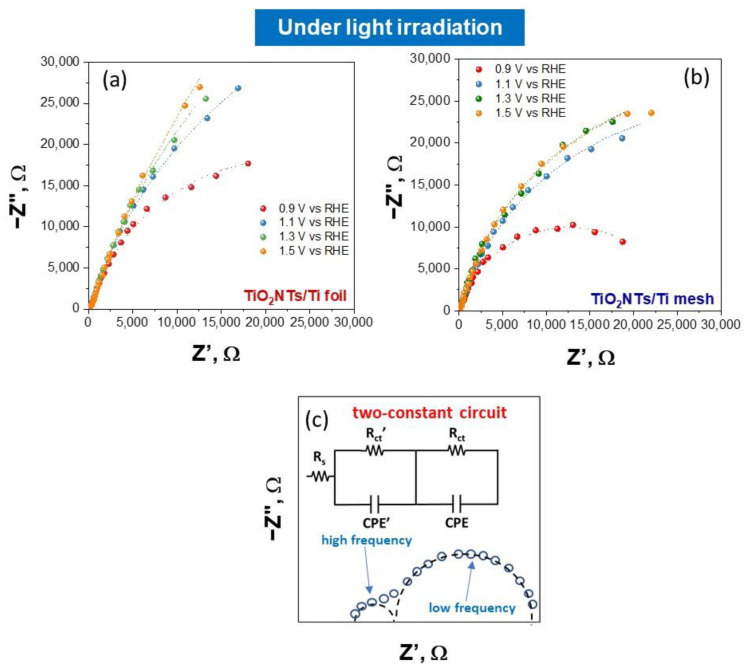
Nyquist plots for TiO_2_NTs/Ti foil (**a**) and TiO_2_NTs/Ti mesh (**b**) measured under light irradiation, varying the applied potential. Filled symbols, experimental impedance data; lines, fitting using the equivalent circuit model (**c**) the two-constant circuit model used to fit all the experimental impedance data.

**Figure 4:**
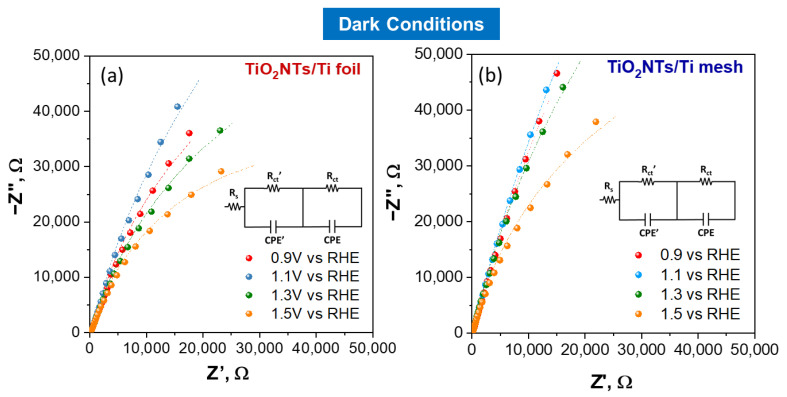
Nyquist plots for TiO_2_NTs/Ti foil (**a**) and TiO_2_NTs/Ti mesh (**b**) measured in dark conditions, varying the applied potential. Filled symbols, experimental impedance data; lines, fitting using the two-constant equivalent circuit model.

EIS analysis was used to investigate the interface resistance of electrodes. The Nyquist impedance plots under light irradiation at different applied potentials are shown in Figure 3a for TiO_2_NTs/Ti foil and Figure 3b for TiO_2_NTs/Ti mesh. The Nyquist plots under dark conditions for the same samples are reported in Figure 4. The experimental data (represented by points) were processed through software simulation of appropriate equivalent circuits (the dashed lines in the graphs refer to the fitting curves). Two-time constants were obtained for all applied potentials, from +0.9 V to +1.5 V vs. RHE, and the equivalent circuit used for the fitting is shown in Figure 3c.

In this circuit, two different charge transfer resistances were considered (Rct’ and Rct), and their presence is better shown in the Bode plots reported in SI (see Figure S8). In detail, the high-frequency Rct’ is usually ascribed to charge transfer phenomena in the liquid phase (such as the ionic migration at the electrode interface [8]), while the Rct (at low frequency) refers to the whole electrochemical process, i.e., both the half-reactions occurring on working and counter electrodes. The applied potential greatly influences the latter, as shown in Figure 3 and Figure 4.

The circuit also consists of a resistor Rs (in series with the parallel circuit consisting of the resistor, Rct and the constant phase element, CPE) related mainly to electrical connections of the cell and the gap between working and reference electrodes in the system. The element CPE is associated with the double layer. All the values resulting from the fitting under light irradiation for TiO_2_NTs/Ti foil and TiO_2_NTs/Ti mesh are listed in Table S1 in SI. The values obtained in dark conditions are reported in Table S2 in SI.

**Figure 5:**
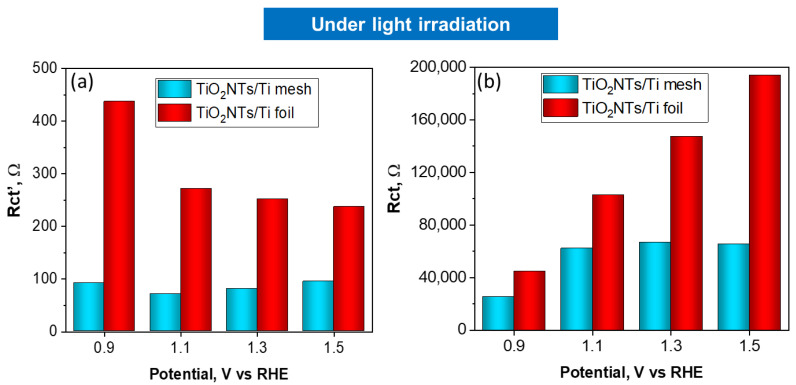
Charge transfer resistances, Rct’ (**a**) and Rct (**b**) obtained by fitting EIS data, vs. applied potential for TiO_2_NTs/Ti foil and TiO_2_NTs/Ti electrodes under light irradiation.

**Figure 6:**
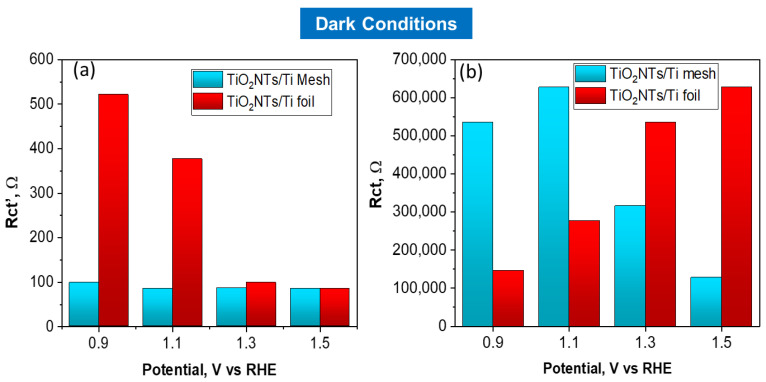
Charge transfer resistances, Rct’ (**a**) and Rct (**b**) obtained by fitting EIS data, vs. applied potential for TiO_2_NTs/Ti foil and TiO_2_NTs/Ti electrodes in dark conditions.

The charge transfer resistances for TiO_2_NTs/Ti foil and TiO_2_NTs/Ti mesh vs. applied potential under light irradiation is reported in Figure 5a,b. The charge transfer resistances vs. applied voltage obtained in the dark are reported in Figure 6a,b. Comparing the charge transfer resistances (Rct) using TiO_2_NT on the mesh or the foil provides useful information about the effect of a 3D vs. 2D meso/nanostructure and the impact on illumination. In fact, TiO_2_NTs/Ti mesh shows a strong diminishing in the resistance under illumination, compared to TiO_2_NTs/Ti foil.

At 1.1 V vs. RHE, the Rct value for the mesh becomes one order of magnitude lower when passing from dark to light conditions, while the effect is much less marked for TiO_2_NTs/Ti foil sample in the same conditions (Rct from 2.77 × 10^5^ ohm in the dark to 1.02 × 10^5^ ohm with light). The high-frequency Rct’ profiles against the applied potential do not show relevant differences in charge transfer resistance under light irradiation, indicating that the resistances associated with ionic migration are negligible in both cases.

EIS experiments were performed also with TiO_2_ P25 as a comparison, and the Niquist plots under light irradiation and in the dark conditions are reported in SI (Figure S9). Comparing the charge transfer resistances obtained in the dark and under light, it is evident that there is a less pronounced effect due to illumination, for this sample.

### 2.4. Photoelectrochemical Testing

The effect of support is investigated in the process of water photo-electrolysis using the three-electrode cell described in Section 3.4., with different electrolytes.

Table 1 shows the current density and hydrogen production rate (normalized considering the different geometrical area of the mesh and foil samples) for TiO_2_NTs/Ti mesh and TiO_2_NTs/Ti foil at three different applied voltages (+2.036, +2.536 and +2.623 V vs. RHE). Results obtained with TiO_2_ P25 are also reported. At all pH values investigated, the structural property of Ti meshes strongly influences the performances.

Specifically, for TiO_2_NTs/Ti mesh at neutral pH (with 0.1 M Na_2_SO_4_), the H_2_ production rate and current density have more than doubled. In the presence of acid/basic electrolyte (in the cathode and anode compartment, respectively), this behaviour is further emphasized with an H_2_ production and current density over three times higher with TiO_2_NTs/Ti mesh, compared to the foil in the same conditions.

When the TiO_2_NTs/Ti mesh was irradiated with light, excited electrons in the valence band (VB) of TiO_2_ were transferred to the conduction band (CB), forming photogenerated electron-hole pairs. The defects, correlated with the 3D geometry of the mesh, promote the transfer of photogenerated holes favouring the evolution of oxygen, while protons and electrons are transferred to the cathode side for hydrogen evolution. Furthermore, the hierarchical porous, round TiO_2_ structure (in the mesh) compared to the planar one (in the foil), offers a large contact area and a faster charge carrier transport, improving the photo electrocatalytic performances [30,31]. Note that the synthesis conditions were individually optimized for TiO_2_NTs foil and mesh, aiming to obtain similar nanotubes with equivalent morphological structure (in terms of length and tube diameter), which can be directly compared. Thus, the strong increase in performances, both in terms of photocurrent and photocatalytic water splitting, observed on TiO_2_NTs mesh when irradiated, cannot be related to aspects such as a relevant change in the thickness or nanomorphology of the TiO_2_NTs layer. On the other hand, in terms of geometrical consideration, on the illuminated area the opposite effect would be expected, e.g., the foil configuration should be preferable. There is also no difference in the composition and purity of the Ti substrate on which the TiO_2_NTs layer is grown. Thus, the possible interpretation is that the curvatures in the wires of the mesh influence the characteristics of the TiO_2_NTs layer.

The CV data clearly indicate that the TiO_2_NTs performances are different from those of pure TiO_2_, and TiO_2_NTs/Ti mesh shows a reduced overpotential in water electrolysis compared to TiO_2_NTs/Ti foil. This is likely associated with creating more defective Ti sites, as suggested by CV and in agreement with diffuse reflectance spectra. Usually, defects favour charge recombination, but CA results indicate a better photocurrent.

Thus, the NTs nanostructure favours the creation of defects responsible for a better photocatalytic water splitting activity, while not markedly influencing the charge separation. These sites are also likely responsible for the enhanced activity in visible light, evidenced by CA experiments.

In addition, EIS results also indicate for TiO_2_NTs/Ti mesh a faster electron transport along the nanotubes towards the metallic non-anodized part, i.e., the internal metallic wires of the gauze. This is also probably related to the strains associated with a change from a rounded to a planar situation, accelerating the electron transfer at the interface.

Furthermore, some studies are reported in the literature but are mainly focused on the effect of an increasing complexity structure and/or growing roughness on the EIS plots. For samples with disorder on a microscopic scale or which are mesoporous, significant variations in the low-frequency region of EIS plot were reported (as observed also in our experiments) and a general deviation from ideal capacitive behaviour directly related to the degree of nanoscale irregularity/roughness of the electrode was also observed [32,33,34,35]. Even if the direct correlation of a three-dimensional geometry on EIS is scarcely investigated, Dyatkin et al. seems to strongly support our hypothesis [36]. The authors investigated the effect of structural disorder on electric conductivity and capacitance for porous electrodes based on carbide-derived carbons and, consistently with our observations, and contrary to the expected, reported that removal of surface defects by annealing decreases the capacitance and forms an obstacle to the movement of ions into pores, thus corroborating our observations. Moreover, the 3D porous architectures with its complexity, can provide noticeable enhancement of the capacitance, increasing the electric field in correspondence to the electrode curvature and surface edges, as reported for conductive carbon nanotubes electrodes [37,38]. Another more recent work clearly evidences, by CV and EIS analysis, the direct correlation of surface defects in MnO_2_ nanosheets, in improving capacitance and decreasing the charge transfer resistances [39], so confirming our hypothesis.

Moreover, the charge transfer resistance, Rct’, is possibly related to an enhanced rate of oxygen bubbles detaching from a round-shaped surface during water splitting with respect to a planar situation.

## 3. Materials and Methods

### 3.1. Chemicals

All reagents and resources were analytically pure and supplied by Merk KGaA, (Darmstadt, Germany). Titanium (Ti) discs (0.025 mm thickness, 35 mm diameter, 99.96% purity) and Ti mesh (80 mesh woven from 0.13 mm diameter) were provided by Alfa Aesar (Lancashire, UK).

### 3.2. Preparation of TiO_2_NTs/Ti Photoanodes

TiO_2_ nanotubes were synthesized on a Ti mesh and on a Ti foil substrate through controlled anodic oxidation [23,40,41].

In detail, before anodization, the metallic supports were cleaned by sonication with isopropanol for 30 min, then dried in air at 100 °C. Then, the clean substrate was placed in a two-electrode electrochemical cell, using an electrolyte based on 2 wt% H_2_O and 0.33 wt% NH_4_F in ethylene glycol under the application of a constant voltage (50 V) for 60 min. A potentiostat (3612A, Agilent Technologies Italia Spa, Cernusco sul Naviglio, MI, Italy) and a multimeter (Keithley 2000, Tektronix, OR, USA) were used to apply a constant potential between the two electrodes and to record the current density. After anodization, the samples were annealed at 450 °C for 3 h (heating rate of 2 °C min^−1^) to generate crystallization of the amorphous TiO_2_ to the anatase phase.

### 3.3. Morphological, Structural and Electronic Characterization

A Phenom ProX Scanning Electron Microscope (SEM) equipped with EDS was used to study the morphology and structure of the TiO_2_NTs-based photocatalysts.

A D2 Phaser Bruker diffractometer equipped with a Ni β-filtered Cu-Kα radiation source was used to investigate the phase composition of the catalysts. In detail, data were collected in a 2θ range from 20° to 60° at a scanning rate of 0.025° s^−1^. The JCPDS database of reference compounds was used to identify the diffraction peaks.

Ultraviolet–visible diffuse reflectance spectra were recorded with a Thermo Fisher Evolution (220) spectrometer with an integrating sphere for solid samples. The light-harvesting characteristics of the photoactive materials were determined using a spectroradiometer (Lot Oriel, model ILT950, Quantum Design Europe, Darmstadt, Germany)

### 3.4. Photoelectrochemical Characterization

The electrochemical characterization (CV, CA) was performed in a three-electrode cell consisting of two compartments (anodic and cathodic) separated by a Nafion N 324 membrane (supplied by Ion Power EU, Munich, Germany). A quartz window allows the irradiation of the catalyst guaranteed by a solar simulator (Lot-Oriel, 300W Xe lamp, Quantum Design Europe, Darmstadt, Germany).

The TiO_2_NTs-based catalyst (a square of dimensions 1 × 1 cm) acted as the working electrode, with a Pt foil (1 × 1 cm) as the counter electrode and a Ag/AgCl (KCl 3M) electrode (supplied by Amel S.r.l., Milano, Italy) as reference. A potentiostat/galvanostat (Autolab pgstat 204, Metrohm Italia S.r.l., Origgio, Varese, Italy) was used to apply the voltage. The potential values referred to the Ag/AgCl electrode are translated to RHE, using the following formula:

E_(RHE)_ = E_(Ag/AgCl)_ + 0.059 pH + 0.21 (1)

All the experimental results are normalized, considering the difference in the geometrical area of the mesh with respect to the foil.

In more detail, CV was performed at a scan rate of 10 mV s^−1^ using two different electrolytes with a continuous flow of N_2_ (5 mL min^−1^) in both compartments of the cell: (i) an aqueous solution of Na_2_SO_4_ 0.1 M as anolyte and catholyte, and (ii) an aqueous solution of KOH 1 M and an aqueous solution of H_2_SO_4_ 0.5 M, as the anolyte and catholyte, respectively.

The CA measurements were performed under ON/OFF light irradiation cycles using KOH 1 M as the anolyte and H_2_SO_4_ 0.5 M as the catholyte, under a continuous flow of N_2_ (5 mL min^−1^). Three different filters were applied to the lamp (AM 1.5G, UVC blocking filter, UVB/C blocking filter) to evaluate the response at different wavelength regions.

### 3.5. EIS Measurements

Electrochemical impedance spectroscopy (EIS) analysis of TiO_2_NTs on Ti mesh and Ti foil was performed in the frequency range from 1 × 10^5^ Hz to 0.01 Hz, with an amplitude of 0.01 Vrms at the applied potential in the range 0.7–1.5 V vs. RHE. The data were collected with and without light irradiation to understand how the light could affect the electrochemical system and the subsequential charge transfer resistances. The electrolyte was Na_2_SO_4_ 0.1 M, with a continuous flow of N_2_ (5 mL min^−1^) in both cell compartments. Impedance data were fitted with Zview^®^ software (Scribner Associates, Southern Pines, NC, USA).

### 3.6. Photoelectrochemical Testing

Photoelectrochemical water-splitting tests were performed using the system described in the Section 3.4. The two compartments of the reactor allow the separated evolution of H_2_ and O_2_. A Gas Chromatographer (7890A, Agilent Technologies Italia Spa, Cernusco sul Naviglio, MI, Italy) equipped with a thermal conductivity detector (TCD) was connected to the cathodic reservoir to measure the amount of H_2_ produced in the water photo-electrolysis reaction. The tests were performed for 90 min using as electrolytes: (i) Na_2_SO_4_ 0.5 M in both compartments and (ii) H_2_SO_4_ 0.5 M (cathodic compartment) and KOH 1 M (anodic compartment). A scheme of the reactor is shown in Figure S10 in SI.

## 4. Conclusions

The results reported in this work evidence that a combination of favourable nanoscale effects determines the enhanced performances of TiO_2_NTs/Ti mesh compared to TiO_2_NTs/Ti foil, contrary to what could be intuitively expected. Accordingly, the structure of the support has a strong impact on the behaviour of the TiO_2_ nanotube array. A good correspondence between EIS and catalytic results in H_2_ production is observed, comparing the resistance values obtained and the catalytic performances. This indicates that EIS is a useful characterization technique allowing better insight into the recombination and charge transfer phenomena and their correlation with the photocatalytic properties.

## Figures and Tables

**Table 1:** Hydrogen production, in µmol h^−1^ cm^−2^ and current density, in mA cm^−2^ for TiO_2_NTs/Ti foil and TiO_2_NTs/Ti mesh samples at different applied voltages and different electrolytes. The formula for calculation of H_2_ production is reported in SI.

Catalyst	Electrolyte *	Applied Potential (V vs. RHE)	H_2_ Production, (µmol h^−1^ cm^−2^)	Average Current Density (mA cm^−2^)
TiO_2_NTs/Ti mesh	Na_2_SO_4_	+2.623 V	24.4	5.08
	H_2_SO_4_-KOH	+2.036 V	9.4	3.03
	H_2_SO_4_-KOH	+2.536 V	182.3	18.69
TiO_2_NTs/Ti foil	Na_2_SO_4_	+2.623 V	10.5	1.88
	H_2_SO_4_-KOH	+2.036 V	5.5	1.16
	H_2_SO_4_-KOH	+2.536 V	46.5	5.24
TiO_2_ P25	Na_2_SO_4_	+2.623 V	0.9	0.21
	H_2_SO_4_-KOH	+2.036 V	0.06	0.14
	H_2_SO_4_-KOH	+2.536 V	4.82	0.63

* Na_2_SO_4_ (0.1 M), H_2_SO_4_ (0.5 M)-KOH (1 M).

## Data Availability

Data available upon request.

## Supplementary Materials

The following supporting information can be downloaded at https://www.mdpi.com/article/10.3390/molecules28083378/s1, Figure S1: XRD patterns of TiO_2_NTs/Ti mesh and Ti foil, Figure S2: Elemental analysis by EDX of TiO_2_NTs/Ti mesh, Figure S3: SEM images of the non-oxidized Ti mesh, Figure S4: SEM image of TiO_2_NTs/Ti foil, Figure S5: UV-visible diffuse reflectance spectra of the TiO_2_NTs/Ti mesh and TiO_2_NTs/Ti foil, Figure S6: Current density vs. time of the TiO_2_NTs/Ti mesh at 1.136 V vs. RHE using open spectrum, Figure S7: Chronoamperometric measurements and Cyclic voltammetry for TiO_2_ P25, Figure S8: Bode plots at different applied potential for TiO_2_NTs/Ti mesh and TiO_2_NTs/Ti foil in dark conditions and under light irradiation, Figure S9: Nyquist plots under light irradiation and dark conditions for TiO_2_ P25, Table S1: Charge transfer resistance parameters data for TiO_2_NTs/Ti foil and TiO_2_NTs/Ti mesh under illumination Table S2: Charge transfer resistance parameters for TiO_2_NTs/Ti foil and TiO_2_NTs/Ti mesh without illumination, Figure S10: Scheme of the PEC reactor and formula for H_2_ production calculation.
