# Supplementary material for: The Role of Substrate Surface Geometry in the Photo-Electrochemical Behaviour of Supported TiO2 Nanotube Arrays: A Study Using Electrochemical Impedance Spectroscopy (EIS)
Source: Molecules. 2023 Apr 11;28(8):3378. doi: 10.3390/molecules28083378 (PMC10142648; doi:10.3390/molecules28083378)
Supplement: Supplementary file 1 [file molecules-28-03378-s001.zip › molecules-2335365-supplementary.pdf]

Supporting information

The role of substrate surface geometry in the photo-electrochemical behaviour of supported TiO_2 nanotube arrays: a study by Electrochemical Impedance Spectroscopy (EIS)

Luana De Pasquale[1], Francesco Tavella[1], Victor Longo[1], Marco Favaro[2], Siglinda Perathoner[1], Gabriele Centi[1], Claudio Ampelli[1] and Chiara Genovese[1,*],

[1] Department of Chemical, Biological, Pharmaceutical and Environmental Sciences – University of Messina, ERIC aisbl and CASPE/INSTM, V.le F. Stagno d'Alcontres, 31, 98166 Messina, Italy

[2] Institute for Solar Fuels, Helmholtz-Zentrum Berlin für Materialien und Energie GmbH, Hahn-Meitner-Platz 1, Berlin, 14109, Germany

Correspondence: chiara.genovese@unime.it

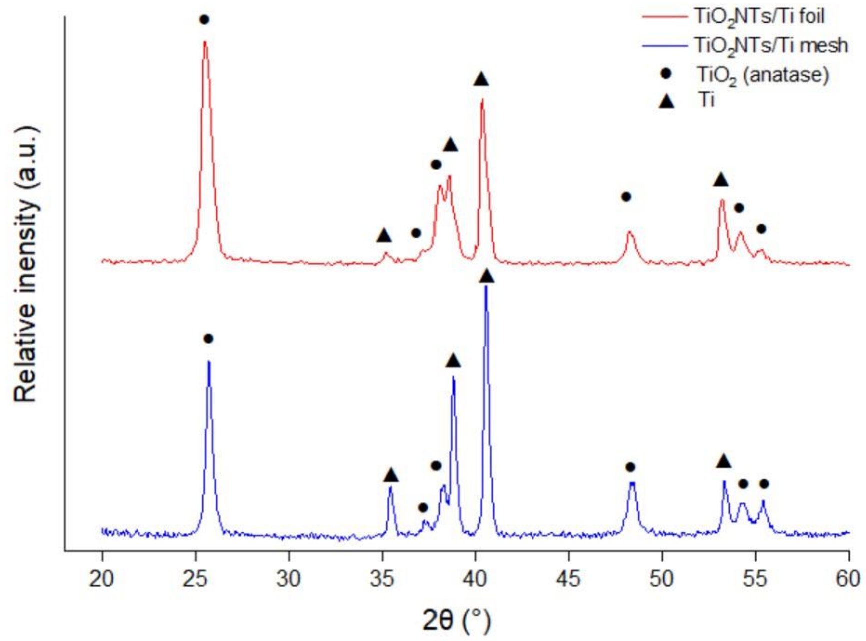

Figure S1. XRD patterns of TiO_2NTs/Ti mesh and Ti foil electrodes.

Element Symbol	Atomic Conc.	Weight Conc.
Ti	24.21	49.28
O	70.77	48.15
C	5.02	2.57

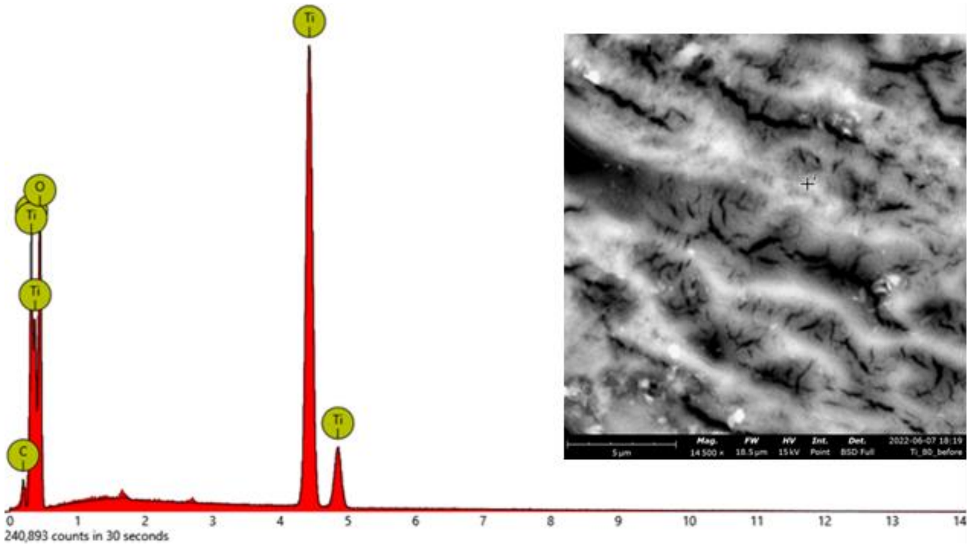

Figure S2. Elemental analysis by EDX of TiO_2NTs/Ti mesh

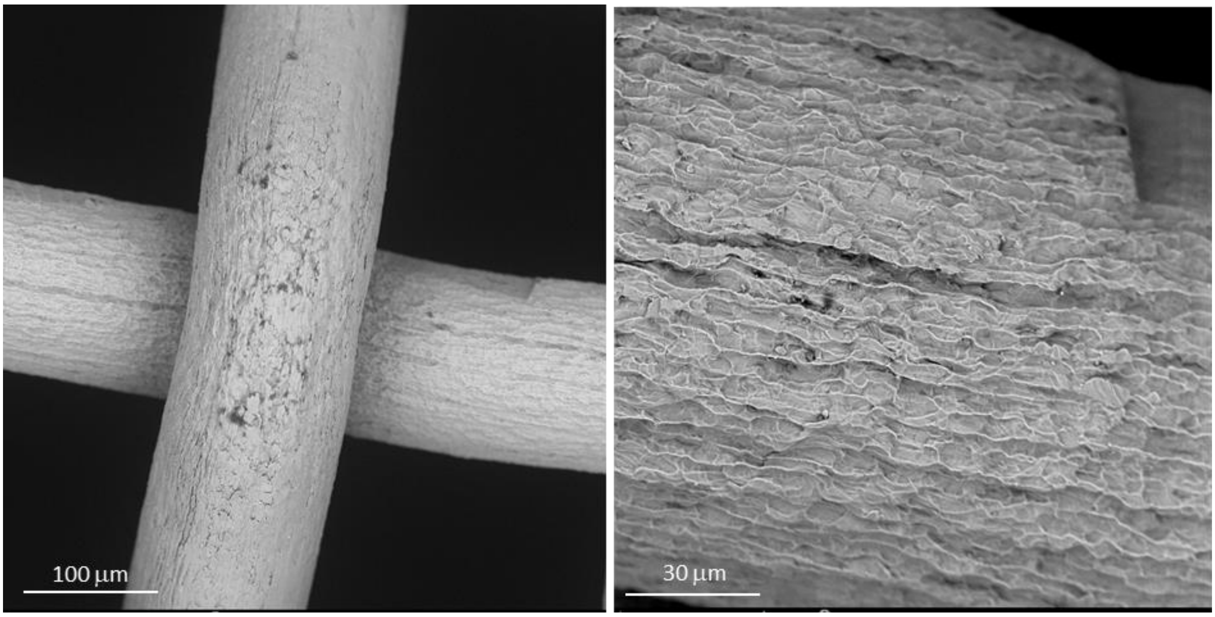

Figure S3. SEM images of the not-oxidized Ti mesh at different magnifications.

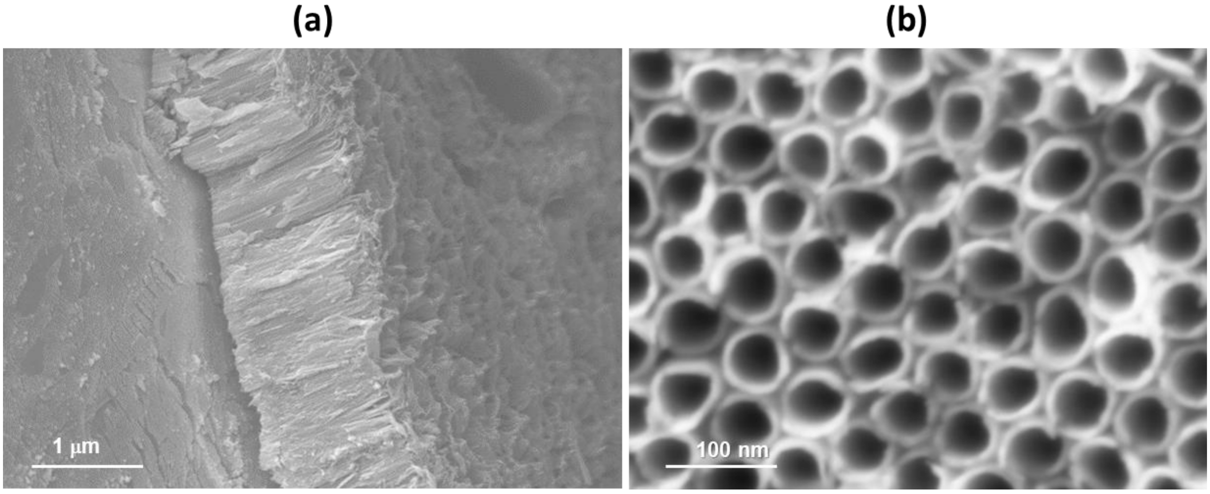

Figure S4. SEM images of cross-section (**a**) and top view (**b**) of the TiO_2NTs on Ti foil.

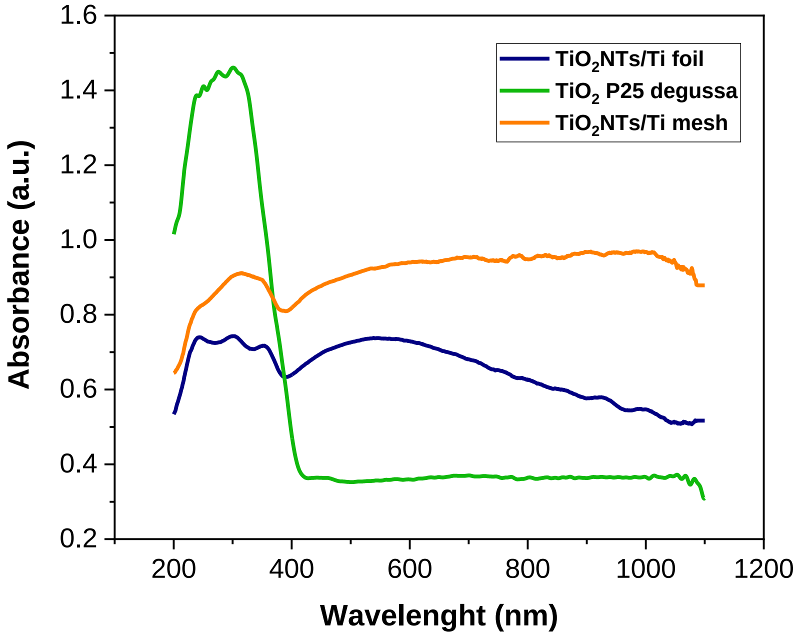

Figure S5. UV-visible diffuse reflectance spectra of the TiO_2NTs/Ti mesh and TiO_2NTs/Ti foil electrodes. The spectrum of TiO_2 P25 (Evonik, former Degussa) is shown for comparison.

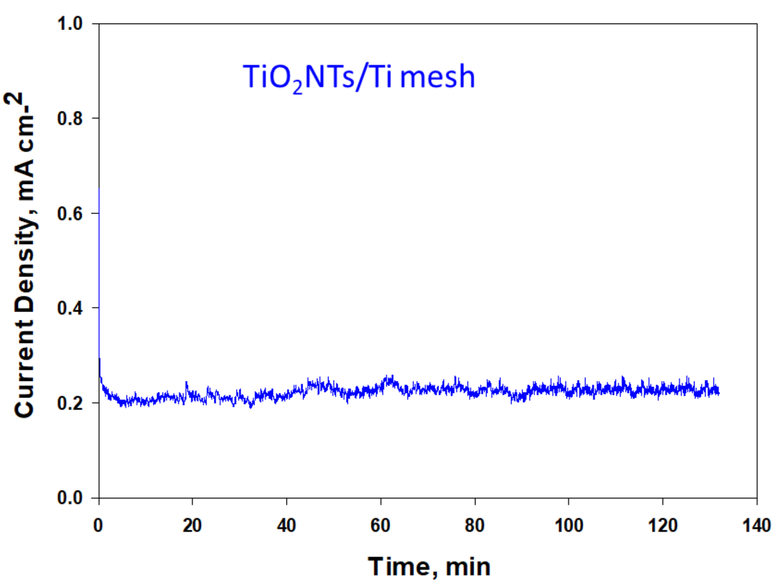

Figure S6. Current density, in mA cm^{-2} vs. time, of the TiO_2NTs/Ti mesh at 1.136 V vs. RHE, in 1 M KOH using open UV-visible lamp spectrum

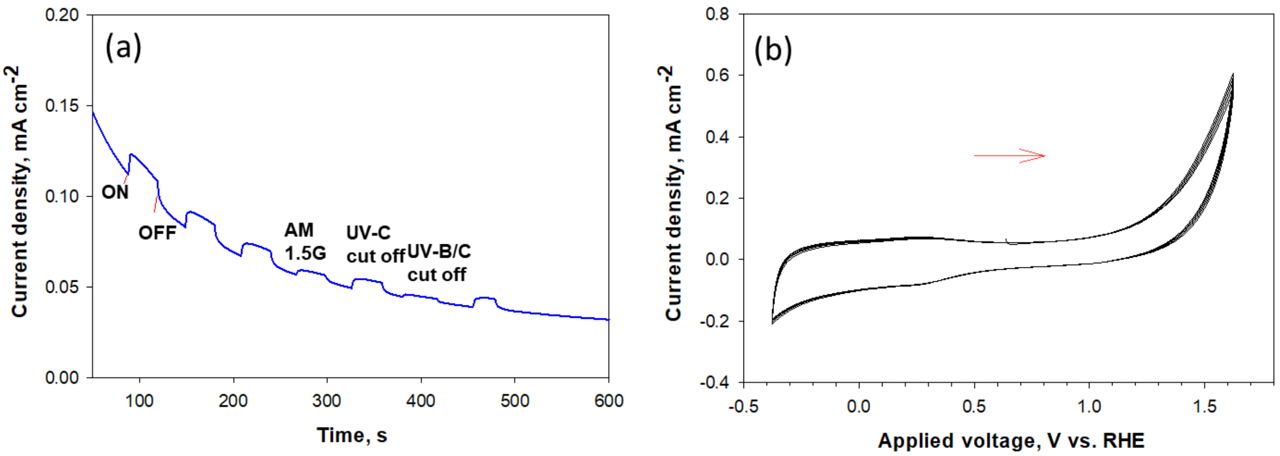

Figure S7. **(a)** Chronoamperometric measurements for TiO_2 P25 at 1.136 V vs. RHE, in 1 M KOH using open UV-visible lamp spectrum (no light filter) and with light filter (AM1.5G, UVC, and UVB/C blocking filter); **(b)** Cyclic voltammetry for the same sample in 1 M KOH. The Ti P25 is deposited on a carbon conductive substrate by spray coating.

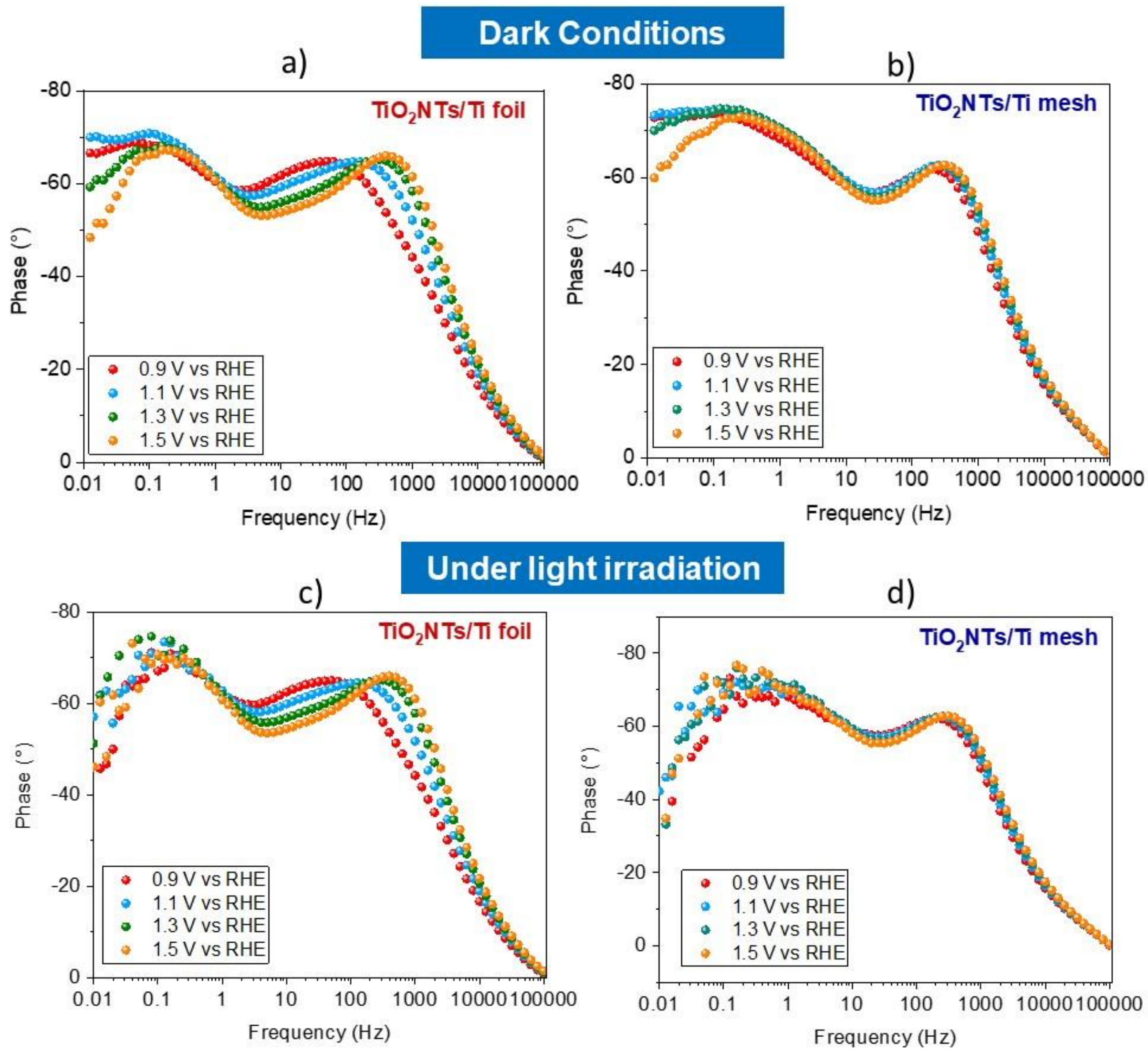

Figure S8. Bode plots at different applied potential for TiO_2NTs/Ti foil and TiO_2NTs/Ti mesh in dark conditions (**a**) (**b**) and under light irradiation (**c**) (**d**).

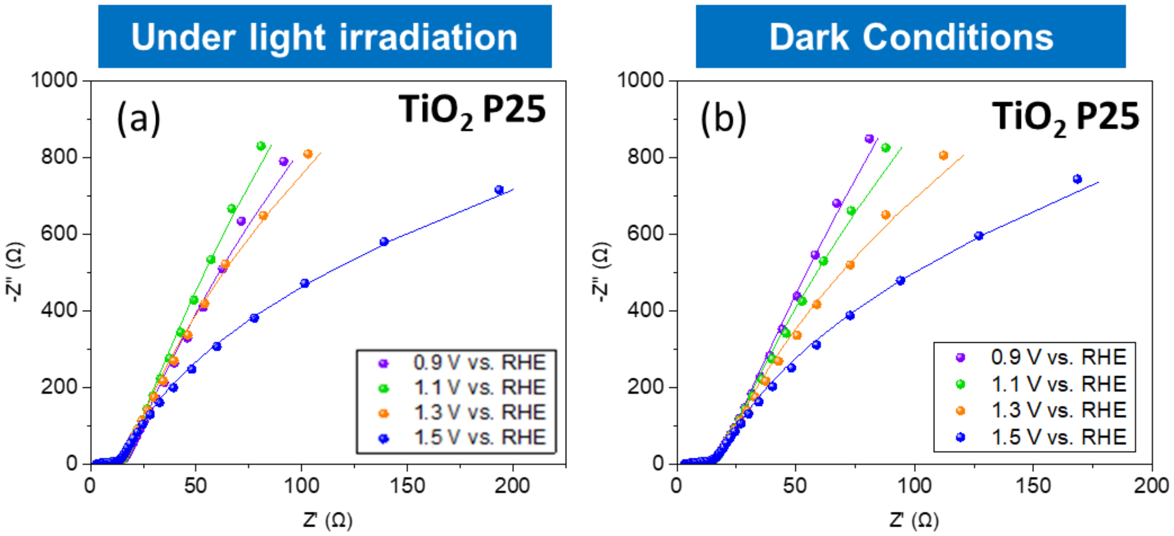

Figure S9. Nyquist plots for TiO_2 P25 measured under light irradiation **(a)** and in dark conditions **(b)** at different applied potential. Filled symbols, impedance experimental data; lines, fitting by using the equivalent circuit model (a two-constant circuit model used to fit all the data).

Table S1. Charge transfer resistance parameters from EIS fitting data for TiO_2NTs/Ti foil and TiO_2NTs/Ti mesh under light irradiation at different applied potentials.

LIGHT IRRADIATION								
	0.9 V vs. RHE		1.1 V vs. RHE		1.3 V vs. RHE		1.5 V vs. RHE	
	TiO_2NTs/Ti foil	TiO_2NTs/Ti mesh	TiO_2NTs/Ti foil	TiO_2NTs/Ti mesh	TiO_2NTs/Ti foil	TiO_2NTs/Ti mesh	TiO_2NTs/Ti foil	TiO_2NTs/Ti mesh
Rs (Ω)	3.87	3.53	3.57	3.52	3.58	3.44	3.57	3.35
Rct′ (Ω)	436.8	91.6	271.7	70.9	251.3	81.0	236.6	94.8
CPE′ (F)	1.74E-04	8.44E-05	5.71E-05	7.42E-05	5.76E-05	6.10E-05	4.36E-05	5.73E-05
Rct (Ω)	4.48E+04	2.53E+04	1.02E+05	6.22E+04	1.47E+05	6.66E+04	1.94E+05	6.53E+04
CPE (F)	3.73 E-04	3.04 E-04	3.38E-04	3.43E-04	3.79E-04	2.87E-04	4.15E-04	2.84E-04

Table S2 Charge transfer resistance parameters from EIS fitting data for TiO_2NTs/Ti foil and TiO_2NTs/Ti mesh without illumination at different applied potentials.

DARK								
	0.9 V vs. RHE		1.1 V vs. RHE		1.3 V vs. RHE		1.5 V vs. RHE	
	TiO_2NTs/Ti foil	TiO_2NTs/Ti mesh	TiO_2NTs/Ti foil	TiO_2NTs/Ti mesh	TiO_2NTs/Ti foil	TiO_2NTs/Ti mesh	TiO_2NTs/Ti foil	TiO_2NTs/Ti mesh
Rs(Ω)	3.83	3.47	3.57	3.34	3.47	3.28	3.34	3.23
Rct′(Ω)	521.0	98.0	376	84.5	98.1	85.3	84.5	84.9
CPE′ (F)	2.57E-05	3.40E-05	2.11E-05	3.28E-05	3.41E-05	2.93E-05	3.27E-05	2.80E-05
Rct(Ω)	1.46E+05	5.35E+05	2.77E+05	6.27E+05	5.35E+05	3.16E+05	6.27E+05	1.28E+05
CPE (F)	9.46E-05	6.74E-05	6.79E-05	6.08E-05	6.76E-05	6.43E-05	6.11E-05	6.29E-05

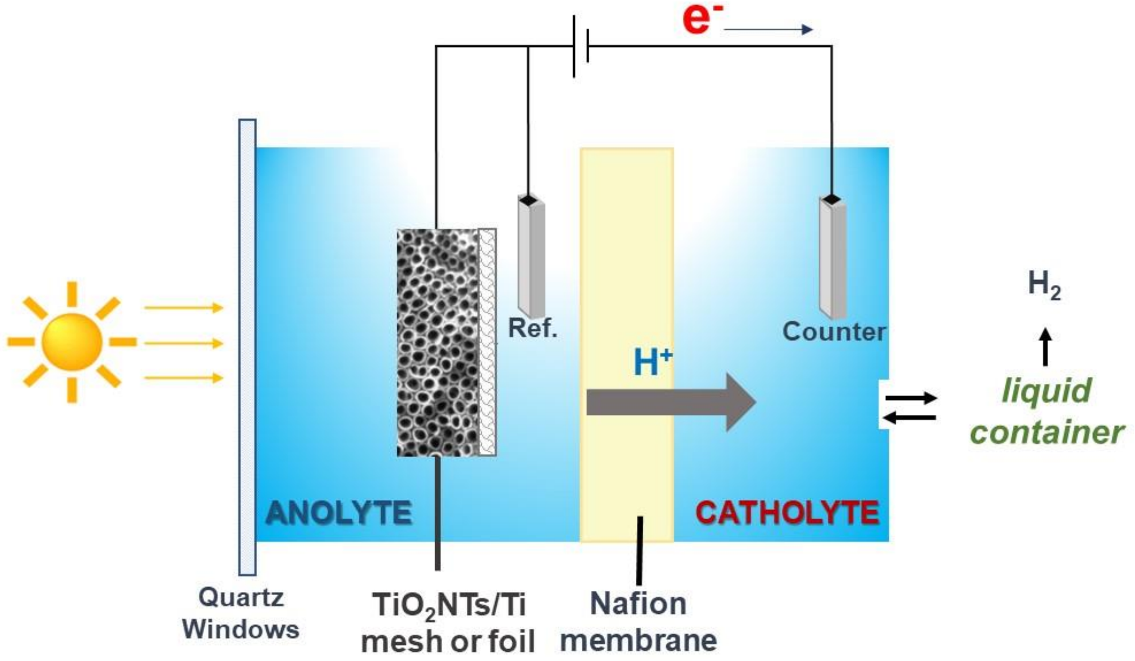

Figure S10. Scheme of the Photo Electro Catalytic (PEC) cell used for water photo-electrolysis.

The formula for calculation of H_2 production is the following:

$$H_2\, production\ (\mu mol \cdot\ h^{-1} cm^{-2}) = \frac{[H_2](\%vol.) \cdot Q\ (L/min)}{V_m\ (L/mol)} \cdot \frac{1}{60\ \cdot A(cm^2)}\ 10^6$$

$[H_2]$ = % of hydrogen detected by the GC

Q = flow rate

V_m = molar volume of a gas at room temperature and atmospheric pressure

A = active area of the catalyst
